# Supplementary material for: 222 nm Far‐UVC from filtered Krypton‐Chloride excimer lamps does not cause eye irritation when deployed in a simulated office environment
Source: Photochem Photobiol. 2023 Apr 27;100(1):137–45. doi: 10.1111/php.13805 (PMC10952573; doi:10.1111/php.13805)
Supplement: Supplementary file 1 — Figure S1. The spectral irradiance of the Far‐UVC lamps used in this study (Biotile, Biocare UV, Warrington, United Kingdom) with both a linear (top) and logarithmic (bottom) y‐axis. [file PHP-100-137-s001.docx]

**SUPPORTING INFORMATION**

# **222 nm Far-UVC from Filtered Krypton-Chloride Excimer Lamps does not Cause Eye Irritation when Deployed in a Simulated Office Environment**

Obaid Kousha^1^, Paul O’Mahoney^2^, Robert Hammond^1^, Kenneth Wood^3^, Ewan Eadie*^4^

1 School of Medicine, University of St Andrews, St Andrews, UK

2 School of Medicine, University of Dundee, Dundee, UK

3 SUPA, School of Physics and Astronomy, University of St Andrews, St Andrews, UK

4 Photobiology Unit, NHS Tayside, Ninewells Hospital and Medical School, Dundee, UK

*Corresponding author email: [ewan.eadie@nhs.scot](mailto:ewan.eadie@nhs.scot) (Ewan Eadie)

**Figure S1.** The spectral irradiance of the Far-UVC lamps used in this study (Biotile, Biocare UV, Warrington, United Kingdom) with both a linear (top) and logarithmic (bottom) y-axis.
